# Mental health and frailty in people with multiple sclerosis: unraveling a complex relationship

**DOI:** 10.3389/fpsyg.2024.1387618

**Published:** 2024-05-22

**Authors:** Nida’ Al Worikat, Anna Zanotto, Jacob J. Sosnoff, Tobia Zanotto

**Affiliations:** ^1^Department of Occupational Therapy Education, School of Health Professions, University of Kansas Medical Center, Kansas, KS, United States; ^2^Department of Occupational Therapy, School of Rehabilitation Science, The University of Jordan, Amman, Jordan; ^3^Department of Physical Therapy, Rehabilitation Science, and Athletic Training, School of Health Professions, University of Kansas Medical Center, Kansas, KS, United States; ^4^Mobility Core, University of Kansas Center for Community Access, Rehabilitation Research, Education and Service, Kansas, KS, United States; ^5^Landon Center on Aging, University of Kansas Medical Center, Kansas, KS, United States

**Keywords:** multiple sclerosis, mental health, frailty, depression, anxiety

## Abstract

People with multiple sclerosis (MS) have up to a 15 times higher risk of being frail compared to age-matched individuals without MS. Frailty is a biological syndrome of decreased physiological reserve and resilience that increases the vulnerability to adverse clinical outcomes and leads to a lower quality of life. Recent studies have begun investigating frailty in the context of MS, highlighting several associations between frailty and adverse events, such as falls, and common MS-related symptoms involving the physical health domain, such as walking and sleeping problems. However, there is a critical knowledge gap regarding the relationship between mental health and frailty in people with MS. This mini-review article aimed to shed light on the potential relationships between MS, frailty, and mental health. Despite the dearth of studies on this topic, indirect evidence strongly suggests that the association between frailty and mental health in people with MS is likely bidirectional in nature. Specifically, mental health disorders such as depression and anxiety may be involved in the etiology of frailty in people with MS. However, they could also be exacerbated by the detrimental effects of frailty on overall health. The complex relationship between frailty and mental health in MS underscores the multifaceted challenges people with MS face. Conducting further research to untangle such a relationship is critical to developing early detection and intervention strategies for improving well-being and medical outcomes in people with MS.

## 1 Introduction

Multiple sclerosis (MS) is a chronic inflammatory disorder that impacts the central nervous system, leading to demyelination of axons, and is considered a primary cause of neurological disability among young adults (Dobson and Giovannoni, 2019). Specifically, the condition is characterized by damage to the myelin sheath surrounding nerve fibers in the brain and spinal cord, interrupting message transmission from the brain to various body parts (Hunter, 2016). People with MS (pwMS) exhibit various symptoms, including sensory, motor, and cognitive impairments, fatigue, and depressive symptoms. These symptoms are likely responsible for the premature onset of frailty often observed in pwMS (Ayrignac et al., 2020). Indeed, previous research has suggested that pwMS have up to a 15 times higher risk of being frail compared to age-matched individuals without MS (Hanlon et al., 2018).

Frailty is a biological syndrome of decreased reserve and resistance to stressors due to the accumulation of deficits across various physiological systems (Clegg et al., 2013). Common frailty symptoms include unintentional weight loss, slow gait speed, muscle weakness, reduced energy, and reduced physical activity. Frailty can lead to a compromised ability to perform activities of daily living and an increased risk of falls, medical complications, hospitalizations, and mortality, consequently decreasing quality of life (Clegg et al., 2013; Veronese et al., 2022). Although frailty is typically considered an age-related condition, several studies suggest that frailty in MS can manifest at a younger age. For instance, Zanotto et al. (2022a) found that approximately two-thirds of pwMS (mean age = 49 years old) living with mild to moderate disability met objective diagnostic criteria for frailty. In addition, Cortese et al. (2022) investigated how MS impacts older women’s health and functioning. The study provided compelling evidence that women who age with MS face an increased susceptibility to frailty at an earlier stage of life compared to women without MS. Particularly, they suggested that MS can accelerate aging by 15–30 years, as their findings indicated that on average, a 45-year-old woman with MS exhibits physical function levels comparable to those of a 75-year-old woman without MS. Several recent studies have explored frailty in MS (Ayrignac et al., 2020; Belvisi et al., 2021; Zanotto et al., 2022a,b). These studies have provided considerable evidence to suggest that frailty may have detrimental effects on the health of people with MS. For example, frailty was found to be strongly associated with adverse clinical outcomes, such as falls (Zanotto et al., 2022a,b), decreased quality of life (Frau et al., 2023), and common MS-related symptoms, such as walking and sleep problems in pwMS (Zanotto et al., 2023; Pradeep Kumar et al., 2024). However, these studies have mainly focused on the physical health aspects of individuals with MS who live with frailty. Currently, there is a critical knowledge gap regarding mental health in the context of frailty in MS.

This mini-review article primarily aimed to shed light on the potential impact of frailty on mental health and vice versa in individuals with MS. Specifically, we aimed to highlight the relationships between MS, frailty, and mental health, ultimately contributing to a more comprehensive understanding of the factors influencing the well-being of pwMS.

## 2 Mental health in people with multiple sclerosis

The unpredictable nature of MS and its physical and cognitive limitations can lead individuals with MS to experience various mental health challenges, including mood disorders such as depression and anxiety (Buhse et al., 2014; Berrigan et al., 2016; Butler et al., 2016; Boeschoten et al., 2017). Depression is a medical condition that affects an individual’s emotions, cognition, and behaviors and is distinguished by a general sense of sadness and an absence of interest in previously pleasurable activities (American Psychiatric Association, 2022). Depression can present various emotional and physical challenges, including impairing an individual’s ability to function effectively at work and in personal life (American Psychiatric Association, 2022). Depression is a significant issue in MS, affecting approximately 50% of patients, a proportion 2–3 times higher than in the general population without MS (Patten et al., 2017). For instance, Yamout et al. (2013) found that depression was one of the strong predictors of overall lower quality of life in pwMS. Depression in MS is influenced by several factors, including elevated stress levels, uncertainty about illness, and inefficient coping strategies (Feinstein et al., 2014). Furthermore, factors closely associated with the effects of the illness, such as lowered enjoyment of recreational activities and difficulties in social relationships, have also been found to contribute to the onset of depression (Feinstein et al., 2014).

Depression in MS is strongly associated with increased morbidity and mortality, including a higher risk of suicide (Feinstein, 2011). In a systematic review of suicidal tendencies in pwMS, Pompili et al. (2012) found that depression was the primary contributing factor to suicidal behavior. In addition, Higuera et al. (2016) have highlighted a relationship between depression and reduced adherence to medication in pwMS, potentially leading to additional MS-related disability. Furthermore, the impact of depressive symptoms on the quality of life in MS is nearly as significant as disability, exerting both direct and indirect effects on overall well-being (Patten et al., 2017). It should also be highlighted that diagnosing depression in the context of MS can be challenging due to the diverse array of symptoms associated with MS. The fifth edition of the Diagnostic and Statistical Manual of Mental Disorders (DSM-5) identifies three specific diagnoses encompassing the range of mood disorders related to multiple sclerosis (MS): adjustment disorder, depressive disorder due to a medical condition, and major depressive disorder (American Psychiatric Association, 2022). In adjustment disorder, depression is seen as a reaction to MS. In contrast, depressive disorder due to a medical condition suggests that depression may result from the inflammatory and degenerative brain changes associated with MS. Ultimately, determining the exact distinction between these diagnoses can be complicated when assessing the patient (Feinstein et al., 2014; American Psychiatric Association, 2022). To diagnose major depressive disorder, the DSM-5 outlines specific criteria, requiring patients to exhibit at least five out of nine well-defined symptoms. However, some of these symptoms, such as fatigue, altered sleep patterns, changes in appetite, poor concentration, and impaired memory (American Psychiatric Association, 2022), may indicate neurological changes associated with MS rather than solely reflective of depressive episodes. This overlap in symptoms can complicate the accurate diagnosis of major depressive disorder in individuals with MS, making it essential for healthcare professionals to carefully consider and differentiate between MS-related neurological changes and depressive symptoms (Feinstein et al., 2014).

Anxiety is another common psychological comorbidity in pwMS (Marrie et al., 2015a). Anxiety disorders are mental health conditions distinguished by excessive worry, fear, and apprehension that persist or worsen over time, and these disorders can significantly impact a person’s daily life and well-being (American Psychiatric Association, 2022). Individuals with MS may experience severe and persistent anxiety due to their worries about the unpredictable nature of future MS episodes and the potential severity of symptoms (Butler et al., 2016). Anxiety disorders are three times more prevalent among pwMS compared to the general population (Silveira et al., 2019). Boeschoten et al. (2017) conducted a systematic review to estimate the prevalence of anxiety in MS and found a high prevalence rate of anxiety that reached 22%. Notably, Generalized Anxiety Disorder emerged as the most prevalent anxiety disorder among pwMS, followed by Panic Disorder, then Obsessive-Compulsive Disorder (Marrie et al., 2015b).

The likelihood of experiencing significant anxiety levels is higher in pwMS who have a greater level of disability, perceived higher levels of general stress, and have lower levels of self-efficacy (Garfield and Lincoln, 2012). Untreated anxiety may lead to issues related to treatment adherence and aggravate symptoms associated with MS (Garfield and Lincoln, 2012). A large population-based study by Marrie et al. (2015a) highlighted a relationship between anxiety and risk of death in nearly 6,000 individuals living with MS. Similarly, studies conducted by Butler et al. (2016) and Korostil and Feinstein (2007) have found correlations between anxiety and an increased likelihood of experiencing suicidal thoughts, as well as a diminished quality of life in pwMS. Additionally, Korostil and Feinstein (2007) found that the development of anxiety disorder in pwMS was significantly associated with being female, having depression as a comorbid, as well as their perceptions of heightened psychosocial stressors and reduced social support.

## 3 Relationship between frailty and mental health

Mental health conditions associated with frailty are often underreported and inadequately understood (Tan et al., 2023). However, several studies from the geriatric literature have revealed a relationship between frailty and the likelihood of developing mental health disorders like depression and anxiety (Mhaolain et al., 2012; Tan et al., 2023). Both conditions can aggravate frailty, resulting in heightened healthcare utilization and diminished functioning (Mutz et al., 2022). Individuals with frailty may have reduced social interactions, limitations in physical functioning, and increased dependency, which can elevate feelings of isolation, sadness, and emotional distress (Mhaolain et al., 2012; Mehrabi and Beland, 2020). Late-life depression and frailty exhibit an overlap, as evidenced by shared symptoms such as weight loss, reduced physical activities, and low energy in depression. In contrast, frailty symptoms include fatigue, decreased leisure activity engagement, and weight loss (Brown et al., 2014). The interplay between frailty and depression has been a subject of recent research. For instance, in their cross-sectional study that included 378 older adults ≥60 years of age with depression diagnosis and 132 without, Collard et al. (2014) found that 27.2% of the participants with depression had frailty while only 9.1% of the non-depressed participants had frailty. At the same time, the depression levels were more severe in the frail participants compared to their non-frail counterparts. While cross-sectional study designs do not allow to infer causality, several studies have postulated that depression may manifest as a result of frailty (Mhaolain et al., 2012; Brown et al., 2014; Soysal et al., 2017), as frailty and ongoing physical deterioration may cause emotional distress (Mhaolain et al., 2012). This distress may encompass sensations of reduced self-worth or hopelessness and culminate in manifestations of depressive symptomatology or, in more severe instances, necessitate therapeutic intervention for depressive disorders (Hladek et al., 2020). From this perspective, promoting self-efficacy coping strategies may represent a valuable strategy to increase resilience and potentially delay the onset of frailty, as Hladek et al. (2020) found that better psychosocial factors were strongly associated with lower odds of being frail in older adults.

Nevertheless, the relationship between frailty and depression is likely bidirectional in nature, and several studies have emphasized the role of depression in developing frailty (Vaughan et al., 2015). The exact mechanisms of how depression may affect overall health and possibly frailty are still unclear. Several factors, such as the use of antidepressant medications, changes in neuronal, hormonal, and immunological function, as well as depression-related social isolation, have been proposed as potential risk factors for frailty (Robertson et al., 2013; Mayerl et al., 2020).

In addition to depression, frail people have a three times higher risk of experiencing anxiety symptoms compared to people living without frailty (Tan et al., 2023). Despite the frequent co-existing with depression, anxiety symptoms are less well documented in the frailty population in comparison to depression. Simultaneously, anxiety and depression tend to be underdiagnosed in this population (Frost et al., 2020). The multifactorial nature of frailty suggests that lifestyle factors like obesity, malnutrition, and low physical activity, all of which are common in pwMS (Veronese et al., 2022), may contribute to disability, leading to anxiety (Tan et al., 2023). For instance, Mhaolain et al. (2012) suggested that the risk of developing clinical depression and anxiety may occur early on in the “lifecycle” of frailty. Indeed, both pre-frail and frail participants in their study experienced more symptoms of anxiety and depression compared to the non-frail. Ultimately, the relationship between anxiety and the decline in physical health, particularly in advanced frailty states, is complex. Real worry and pathological anxiety may likely arise due to concurrent physical illnesses. Conversely, chronic anxiety might also play a role in the deterioration of health, cognitive capacities, and functional abilities, potentially contributing to heightened morbidity and mortality and acting as a primer for frailty (Mhaolain et al., 2012; Gulpers et al., 2016; Margioti et al., 2020). This aligns with the findings of Mutz et al. (2022) in their longitudinal analysis, indicating that individuals with anxiety have a higher likelihood of developing frailty than people without anxiety, with females exhibiting higher levels of frailty compared to males. Notably, many frailty conceptualizations include psychological and social aspects (Gobbens and van Assen, 2014), which underscores the intrinsic relationship between frailty and mental health.

Despite the lack of evidence concerning the relationship between frailty and mental health in pwMS, a recent study by Frau et al. (2023) investigated the quality of life of pwMS living with or without frailty. The study highlighted a significantly lower quality of life in the frail compared to non-frail participants using the Multiple Sclerosis Impact Scale-29. This assessment tool encompasses both physical and psychological aspects of quality of life. While these findings provide the first indirect evidence of a relationship between frailty and mental health in pwMS, the mechanisms linking these two factors remain largely unexplored. Interestingly, Pradeep Kumar et al. (2024) found a significant association between frailty and worse sleep quality in pwMS in a cross-sectional study involving 76 pwMS. Sleep problems are common and linked to lower quality of life in pwMS (Laslett et al., 2022). Analogously, sleep disturbances are highly associated with the occurrence of mental health disorders (Freeman et al., 2020), as an insufficient amount of sleep is known to increase anxiety levels (Palmer and Alfano, 2020). Therefore, it is plausible that poor sleep quality may mediate the bidirectional relationship between frailty and mental health in pwMS. In other words, it is possible that frail individuals may have more difficulty sleeping, which in turn may aggravate mental health issues (Baione et al., 2023) or vice versa. However, it is also possible that interactions between mental health and sleep problems may accelerate the vicious cycle of frailty in pwMS.

## 4 Conclusion

In this mini-review article, we focused on mental health, specifically depression, and anxiety, a critical component of overall health yet often overlooked. To the best of our knowledge, no research studies have yet been conducted to examine the mental health status of individuals with MS living with frailty and investigate the potential role of frailty on the mental health of pwMS. However, based on this brief preliminary review of the literature and the complex interrelationships between MS, frailty, and mental health, it is plausible that both MS and frailty can individually affect mental health, which highlights the multifaceted nature of the challenges faced by pwMS. This presents a promising area for further investigation. Specifically, untangling the relationship between mental health and frailty could lead to identifying novel strategies for promoting well-being and quality of life in pwMS by allowing early detection and intervention, stemming from a better understanding of this relationship.

Last but not least, there is a crucial need to emphasize multidimensional assessment approaches in routine clinical evaluations and comprehensive multidisciplinary interventions that target both the physical and psychological well-being of pwMS living with frailty. This could be critical in preventing the escalation of health-related problems, ultimately leading to improved medical outcomes.

## Author contributions

NA: Writing – original draft, Writing – review & editing. AZ: Writing – review & editing. JS: Writing – review & editing. TZ: Writing – review & editing.
